# Mortality After Operatively Treated Cervical Spine Fractures – A National FinSpine Register Study

**DOI:** 10.1177/21925682261442458

**Published:** 2026-04-15

**Authors:** Nils Danner, Nikolai Klimko, Henri Salo, Ville Leinonen, Jukka Huttunen

**Affiliations:** 1 Spine Center and Neuro Center, Department of Neurosurgery, 60650 Kuopio University Hospital, Kuopio, Finland; 2 Institute of Clinical Medicine, University of Eastern Finland, Kuopio, Finland; 3 3837 Finnish Institute for Health and Welfare, Helsinki, Finland

**Keywords:** cervical spine, fracture, surgery, mortality, register

## Abstract

**Study Design:**

Nationwide register study.

**Objective:**

The incidence of cervical spine fractures and related surgeries are increasing in the ageing population, yet population-based outcome data remain limited. This study aimed to identify factors associated with mortality and to analyze causes of death following surgical treatment for cervical spine fractures in a nationwide cohort.

**Methods:**

A cohort of 979 patients undergoing surgery for cervical spine fractures (2017-2024) was identified from the nationwide FinSpine register. Independent risk factors for mortality were identified with Cox proportional hazards models. Patient data were linked with national records on mortality and causes-of-death. Standardized mortality ratios (SMRs) were calculated for comparison with the age- and sex-matched general population.

**Results:**

The 1-year mortality rate was 11.8%, increasing with age from 3.9% in patients younger than 65 years to 24.9% in those aged 85 years or older. Increasing age (HR 1.06 per year) and spinal cord injury (HR 1.71) predicted mortality. Compared with the general population, mortality was significantly elevated across all age groups, with highest standardized mortality ratio in patients younger than 65 years (SMR 8.3) and lowest for patients aged ≥85 years (SMR 2.0). External causes (accidents and violence) were the leading causes of death and highly over-represented (SMR 22.7) in the patients.

**Conclusions:**

Increasing age and spinal cord injury predict mortality after surgery for cervical spine fractures. The low relative excess mortality in elderly patients, compared to the general population, supports the potential role of operative management with careful patient selection even in advanced age.

## Introduction

The incidence of cervical spine fractures is increasing with an ageing population, and the typical trauma mechanism has shifted from high-energy injuries in the young to low-energy falls in the elderly.^1-7^ The prognosis of elderly patients with cervical spine fractures is regarded as poor, and the mortality rates range from 3% within the first month to over 30% within the first year after the injury, regardless of whether patients are treated conservatively or operatively.^7-13^ A benefit of surgical treatment has been shown in terms of reducing mortality in upper cervical spine injuries, but selection bias may have affected the choice of treatment.^14-16^ Furthermore, most studies have been conducted in selected patient populations, and there is a lack of population-based outcome data after operative treatment of cervical spine fractures. National registries have gained increasing popularity for studying outcomes of spine surgery especially in the Nordic countries, where publicly funded and operated health care systems provide an ideal setting to study the outcomes of spine surgery on a population-based level. In this study, we aimed to investigate factors affecting mortality after surgically treated cervical spine fractures in a nationwide cohort.^17^

## Materials and Methods

The Finnish public healthcare system is tax-funded and equally accessible for all citizens, ensuring a national population-based coverage. The Finnish spine register (FinSpine) is a national quality register curated by the Finnish Institute for Health and Welfare.^17^ FinSpine was implemented in 2016 and currently it collects data on all spine surgeries performed in the country. The register is used by all Finnish public hospitals and major private hospitals, including all centers which perform cervical spine trauma surgery. For the current study FinSpine data was augmented with data from Statistics Finland (public statistics authority) on mortality and causes of death for the patients as well as, for the general population.^18^ Data on the use of opioids was retrieved from the Finnish Social Insurance Institution.

Patients for the current study were identified from FinSpine by the timing of surgery between January 5^th^ 2017 and February 29^th^ 2024, the exact dates being determined by database dumps. The follow-up time was until death or until the end of the study period. The following parameters were used for the statistical analyses: time of surgery, time of death, age, sex, spinal cord injury, perioperative complications, level of surgery (occipitocervical vs upper cervical (C1-2) vs sub-axial), type of surgery and opioid use (before and after surgery). The type of surgery was divided into 4 categories: anterior (anterior cervical decompression and fusion, ACDF or corpectomy), odontoid screw fixation, posterior (pedicle screw and/or lateral mass fixation) or 360-degree fixation. Patients whose type of surgery was recorded as other or who underwent decompression alone were excluded from the outcome analyses on the type of surgery. In Finland, sex assigned at birth is recorded in national registries at the time of birth and associated with an individual life-long personal identification number, which allows the identification of every individual. Gender, race and ethnicity are not recorded in national registers. Statistical analyses were performed with IBM SPSS Statistics (software version 29.0). Kaplan-Meier survival curves were calculated separately for different age groups, the presence of spinal cord injury and the type of surgery. Group differences were compared with one-way ANOVA for continuous data and Pearson's chi square for categorical data. Furthermore, a Cox regression analysis was carried out with the following variables: age, sex, pre- and postoperative opioid use, perioperative complications, level(s) of surgery, spinal cord injury and type of surgery. Cox regression analyses were performed only among patients with complete data for all included covariates.

In Finland, by law, all deaths of persons with permanent Finnish residence must be reported by physicians using a standardized death certificate. Each certificate records the date of death, the underlying, immediate and contributory causes, the place and manner of death as well as the method used to establish the cause of death.^18^ The archive is curated by Statistics Finland and can be used for research purposes. For the FinSpine cohort of surgically treated cervical-spine-fracture patients, cause-specific mortality data were available until December 31^st^ 2023. This date was used as the censoring point for all cause-specific mortality analyses. For the general Finnish population, mortality rates and causes of death (all-cause and cause-specific) were retrieved in aggregated form from the publicly accessible Statistics Finland database from 2017 to 2023. Causes of death are classified in accordance with ICD-10 (International Classification of Diseases - 10th edition). Specific trauma diagnosis codes (ICD-10 S00–S99), which detail the anatomical site and nature of injury, are not reported in the datasets provided by Statistics Finland but all injury‐related deaths are classified by their external cause codes (ICD-10 V01–Y98). Standardized mortality ratios (SMRs) were calculated by direct standardization, applying the sum of annual population death rates from 2017 to 2023 stratified by age band, sex and underlying cause of death. Confidence intervals of 95% were derived by the Poisson method. To align the FinSpine cohort with the annual reference data, for SMR calculations, follow-up in the FinSpine cohort was restricted to complete calendar years (2017-2023).

The use of the registers applied in the current study has been permitted for research purposes by the Finnish Institute for Health and Welfare and approved by the Ministry of Social Affairs and Health of Finland (THL/216/6.02.00/2024). According to Finnish research legislation, register-based studies do not require separate institutional ethical board approval.

## Results

**Figure 1.**
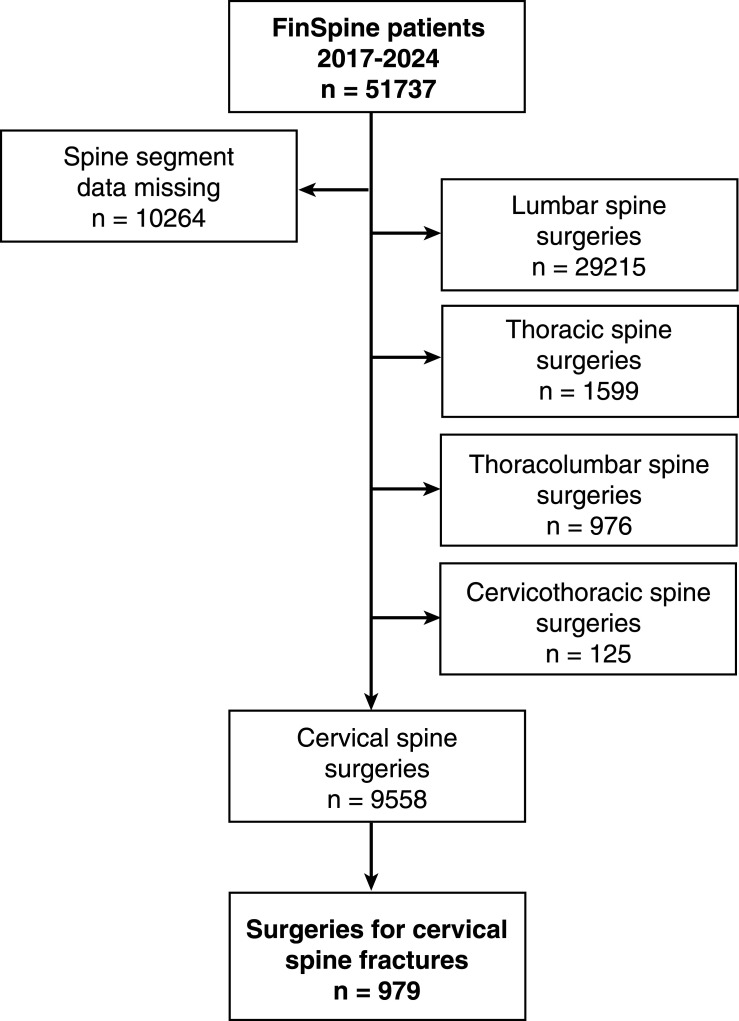
Flow chart of the study

The flow chart of the study is presented in Figure 1. 979 patients (74.7% male and 25.3% female) who underwent cervical spine surgery due to a fracture formed the study population. Demographic and surgical data are provided in Table 1. The mean age of the patients was 64 ± 18 years (range 15 to 95 years). The mean follow-up time was 35.2 ± 22.0 months (Mean ± SD). Posterior fixation accounted for 55.1% (n = 532) of operations, anterior approaches (ACDF or corpectomy) for 28.1% (n = 271), combined approaches (360-degree) for 4.0% (n = 39), odontoid screw fixation for 3.0% (n = 29), and other procedures for 9.8% (n = 95).

**Table 1.** Patient Demographics and Comparison Between Outcome Groups

	All patients	Deceased	Alive	*P*-value
Number of patients, n	979	209	770	
Age, years (mean ± SD)	64 ± 18	74 ± 12	61 ± 18	<0.001
Sex; male, n (%)	731 (74.7)	156 (74.6)	575 (74.7)	
Spinal cord injury, n (%)	255 (28.8)	76 (41.5)	179 (25.5)	<0.001
Complication, n (%)	37 (3.8)	10 (4.8)	27 (3.5)	
Fusion level, n (%)				0.036
Occipitocervical	42 (4.5)	14 (7.3)	28 (3.8)	
Subaxial	764 (81.4)	146 (75.6)	618 (82.8)	
Upper cervical	133 (14.2)	33 (17.1)	100 (13.4)	
Opioid use postoperatively	343 (35.0)	42 (20.1)	301 (39.1)	<0.001
Type of surgery				<0.001
Anterior (ACDF or corpectomy)	271 (28.1)	31 (15.0)	240 (31.6)	
Posterior fixation	532 (55.1)	142 (68.6)	390 (51.4)	
360 fixation	39 (4.0)	7 (3.4)	32 (4.2)	
Odontoid screw fixation	29 (3.0)	3 (1.4)	26 (3.4)	
Other	95 (9.8)	24 (11.6)	71 (9.4)	

Percentages are reported within groups.

Data on spinal cord injury, fusion level, and type of surgery were available for 884 (90.3%), 939 (95.9%), and 966 (98.7%) patients, respectively.

Group differences were assessed using the independent-samples t-test and the chi-square test.

Only significant *P*-values (*P* < 0.05) are shown.

Opioid use was determined as purchases of opioids after surgery.

ACDF, anterior cervical decompression and fusion.

At the end of the study period 770 patients were alive and 209 had deceased. Outcome data and specific demographics in different age groups are shown in Table 2. The overall mortality rate at 1 year after surgery was 11.8%, ranging from 3.9% in patients <65 years to 24.9% in patients ≥85 years of age. Excess mortality was observed in patients of all age groups as compared to the general Finnish population. The SMRs ranged from 2.0 (95% CI 1.4-2.8) for patients aged ≥85 up to 8.3 (95% CI 5.8-11.6) for those aged <65 years.

**Table 2.** Comparison of Patients Between Different Age Groups

Age	<65 years	65-74 years	75-84 years	≥85 years	*P*-value
Number of patients, n	363	301	246	69	
Sex; male, n (%)	285 (78.5)	235 (78.1)	165 (67.1)	46 (66.7)	0.002
Spinal cord injury, n (%)	93 (28.9)	78 (27.8)	68 (31.2)	16 (25.4)	0.774
Complication, n (%)	11 (3.0)	16 (5.3)	10 (4.1)	0 (0.0)	0.152
Fusion level, n (%)					<0.001
Occipitocervical	8 (2.3)	21 (7.3)	6 (2.5)	7 (10.6)	
Upper cervical	37 (10.8)	52 (18.1)	35 (14.5)	9 (13.6)	
Subaxial	299 (86.9)	214 (74.6)	201 (83.1)	50 (75.8)	
Opioid use postoperatively	129 (35.5)	114 (37.9)	86 (35.0)	14 (20.3)	0.052
Type of surgery					<0.001
Anterior (ACDF or corpectomy)	158 (44.0)	61 (20.5)	38 (15.8)	14 (20.6)	
Posterior fixation	130 (36.2)	188 (63.1)	167 (69.3)	47 (69.1)	
360 fixation	22 (6.1)	10 (3.4)	6 (2.5)	1 (1.5)	
Odontoid screw	6 (1.7)	11 (3.7)	10 (4.1)	2 (2.9)	
Other	43 (12.0)	28 (9.4)	20 (8.3)	4 (5.9)	
Mortality, n (%)					<0.001
1 month	1.4	1.7	8.5	11.6	
3 months	2.2	3.0	10.6	14.5	
1 year	3.9	7.1	14.2	24.9	
2 years	6.9	10.6	23.0	41.3	
3 years	10.6	14.0	31.2	47.7	
SMR (CI 95%)	8.3 (5.8-11.6)	3.0 (2.2-3.9)	3.1 (2.4-3.9)	2.0 (1.4-2.8)	

Percentages are reported within groups.

Data on spinal cord injury, fusion level, and type of surgery were available for 884 (90.3%), 939 (95.9%), and 966 (98.7%) patients, respectively.

*P*-values were obtained using the chi-square test for categorical variables and the log-rank (Mantel–Cox) test for overall differences between survival groups.

Opioid use was determined as purchases of opioids after surgery.

Standardised mortality ratios (SMRs) were calculated by direct standardisation using annual national population death rates stratified by age and sex. For SMR calculations, follow-up in the cervical spine fracture cohort was limited to complete calendar years (2017-2023).

ACDF, anterior cervical decompression and fusion; CI 95%, 95% confidence interval.

**Figure 2.**
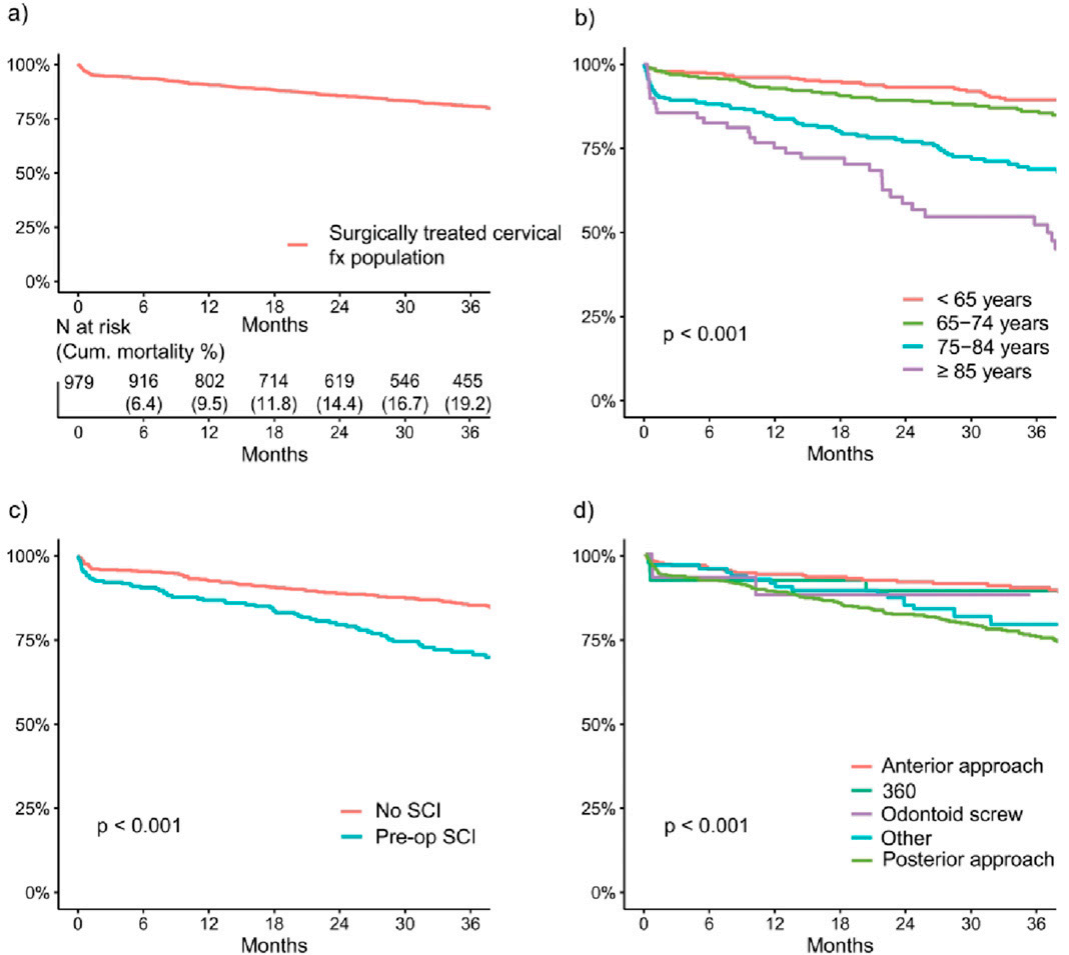
Kaplan-Meier curves for survival. Kaplan-Meier curves are presented for the entire population (A) as well as for all independent risk factors for mortality (B-D)

Kaplan-Meier survival curves are presented in Figure 2. The Cox proportional hazards model (Table 3) showed that age (HR = 1.06 per year, *P* < 0.001) and spinal cord injury (HR = 1.71, *P* = 0.001) were independent risk factors for mortality. Patients who were treated with posterior surgery had a higher risk of mortality as compared to anterior surgery (HR = 1.76, *P* < 0.05). Sex, level of surgery, complications and pre- or postoperative opioid use did not influence mortality.

**Table 3.** Independent Risk Factors for Mortality After Operatively Treated Cervical Spine Fracture

Covariate	Hazard ratio	CI 95%	*P*-value
Age	1.06	1.04-1.07	<0.001
Sex (female)	0.90	0.63-1.29	0.571
Pre-operative opioid purchases (yes)	1.26	0.46-3.46	0.661
Post-operative opioid purchases (yes)	0.46	0.14-1.45	0.184
Pre-operative strong opioid purchases (yes)	0.95	0.31-2.92	0.930
Post-operative strong opioid purchases (yes)	1.21	0.37-4.01	0.749
Perioperative complication (yes)	1.19	0.55-2.56	0.658
Pre-operative spinal cord injury	1.71	1.24-2.37	0.001
Fusion level			
Subaxial - reference	1		
Upper cervical (C1-2)	1.05	0.67-1.63	0.844
Occipitocervical	1.20	0.64-2.26	0.572
Type of surgery			
Anterior - reference	1		
Posterior	1.76	1.11-2.79	0.017
360-degree	1.16	0.47-2.86	0.752
Odontoid screw	1.63	0.48-5.48	0.430
Other	1.35	0.71-2.58	0.363

The Cox proportional hazards regression analysis was conducted on 842 patients (86.0%) with complete data. Covariates were analysed using the Enter method. ACDF, anterior cervical decompression and fusion; CI, 95% confidence interval.

**Figure 3.**
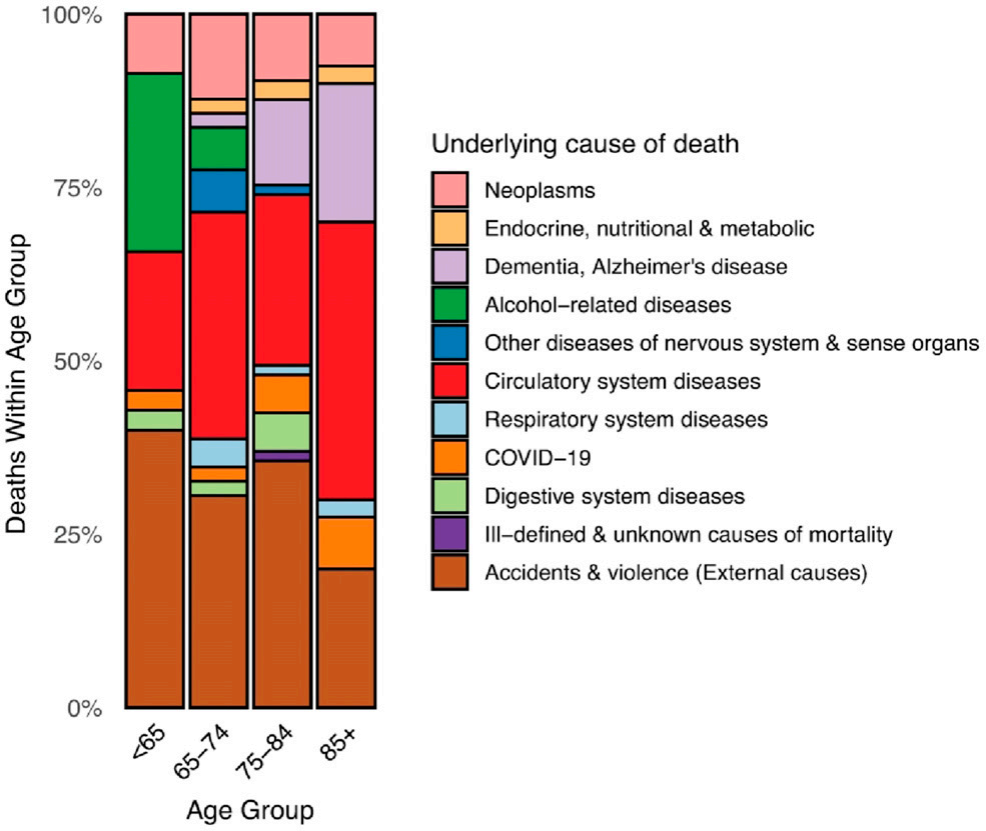
Distribution of causes of death by age group for patients operated due to cervical spine fractures

Cause-specific mortality data is presented in Table 4. The most frequent causes of death were external causes and diseases of the circulatory system. Compared with the general Finnish population, the patients had elevated SMRs for most cause-specific mortality categories. For deaths by external causes, the SMR was 22.7 (95% CI 17.4-29.0). Within the external-cause category, falls accounted for over half of deaths, followed by long-term sequelae of external causes and transport accidents. Among the 63 external cause fatalities, the overall median interval from surgery to death was 32 days (mean 270.7 days, range 0-2070 days). The distribution of causes of death within different age groups is shown in Figure 3. External causes predominated in all but the oldest age group, whereas circulatory diseases were the most common cause of death in patients aged ≥85 years. Alcohol-related fatalities declined with increasing age, representing 25.7% in the youngest age group and not observed after the age of 74. The proportion of deaths due to dementia and Alzheimer’s disease rose with increasing age up to 20.0% in the oldest age group.

**Table 4.** Cause-Specific Mortality

Cause of death	Study cohort		General population		SMR	CI 95%
	n	%	n	%		
Neoplasms	19	9.6	93 449	23.4	1.4	0.8-2.2
Endocrine, nutritional and metabolic	4	2	6029	1.5	13.7	3.7-35.1
Dementia, Alzheimer’s disease	18	9.1	76 258	19.1	2.4	1.4-3.8
Alcohol-related diseases and alcohol poisoning	12	6.1	11 712	2.9	14.9	7.7-26.0
Other diseases of the nervous system and sense organs	4	2	14 410	3.6	5.0	1.4-12.8
Circulatory system diseases	57	28.9	132 331	33.1	2.8	2.1-3.6
Respiratory system diseases	4	2	13 855	3.5	3.5	0.9-8.9
COVID-19	9	4.6	8088	2	8.8	4.0-16.6
Digestive system diseases	6	3	9220	2.3	9.4	3.4-20.4
Ill-defined or unknown causes of death	1	0.5	1650	0.4	29.3	0.7-163.2
External causes (accidents and violence)	63	32	22 465	5.6	22.7	17.4-29.0
Falls	35	17.8	8641	2.2		
Intentional self-harm	2	1	5336	1.3		
Sequelae of external causes	12	6.1	3001	0.8		
Transport accidents	11	5.6	1711	0.4		
Contact with heat or hot substances	1	0.5				
Event of undetermined intent	1	0.5				
Exposure to forces of nature	1	0.5				
Total	197	100				

Cause-specific mortality among national spine surgery register patients (n = 979) undergoing surgical treatment for cervical spine fractures between 23 January 2017 and 31 December 2023 was compared with that of the general population over the same period. Cause-specific data were available until 31 December 2023; accordingly, follow-up was censored at that date, and any subsequent deaths were excluded. Cumulative all-cause mortality during this interval was 20.1% (n = 197) in the cervical spine fracture cohort vs 7.2% in the general population. Standardised mortality ratios (SMRs) were calculated by direct standardisation using annual national population death rates stratified by age, sex and underlying cause of death. CI, 95% confidence interval.

## Discussion

This is one of the very few studies worldwide addressing the outcome of cervical spine fracture surgery in a national population-based setting. The main advantage of the current study is the use of the nationwide prospective FinSpine register. The register covers all units, which perform surgery for spine injuries in Finland. Due to a publicly funded healthcare system, there is no selection bias due to socioeconomic, insurance-related or compensatory issues and the choice of treatment is done solely on a medical basis. Furthermore, mortality data is collected by Statistics Finland, which has complete coverage for all people with permanent Finnish residence or nationality.

We found that the overall mortality rate at 1 year after surgery was 11.8%. Mortality increased with age, ranging from 3.9% in patients younger than 65 years to 24.9% in those aged 85 years or older (Table 2). To evaluate excess mortality, we compared our study population with the general population of the same age groups. According to Statistics Finland, in 2023 the 1-year mortality in the general population ranged from 0.21% in people under 65 years of age to 15.9% in people aged 85-95 years. The mortality rate is lower than usually reported in this age group, but also in literature there seems to be a trend towards better survival in more recent studies.^8,10,13,19-21^ Patients who had undergone surgery for cervical spine injuries had higher mortality rates in all age groups as compared to the general population. Interestingly, the excess mortality was highest in the younger patients, and the SMR decreased with age. The highest SMR, 8.3 (95% CI 5.8-11.6) was observed in patients aged <65 years, whereas in very old patients (≥85 years) the SMR was only 2.0 (95% CI 1.4-2.8).

Trauma-related causes of death were highly over-represented in the patients operated due to cervical spine fracture as compared to the general population, with an SMR 22.7 (95% CI 17.4-29) for deaths by external causes (Figure 3 and Table 4). These numbers include deaths due to the initial injury as well as possible subsequent traumatic deaths. With the exception of respiratory system diseases and neoplasms also all other recorded causes of death were over-represented in the patients as compared to the general population. The all-cause excess mortality is most likely explained by the aggravation of underlying diseases after a traumatic event even if the trauma is not the cause of death per se. This can also be seen in the distribution of causes of death in the patients (Figure 3), where the proportion of traumatic and alcohol-related causes of death decrease with age, and conversely, the proportion of dementia and circulatory system diseases increase with age.

FinSpine includes only operatively treated patients, and therefore a comparison with conservative treatment was not performed. The selection of surgical candidates, especially in the elderly population, is very likely to be influenced by the general health and functional ability of the patients. Accordingly, there is an unquestionable selection bias favouring surgery in patients with better general health as opposed to those who are assigned to conservative treatment.

Since patients with severe comorbidities, which increase surgery-related risks, are more likely to be treated conservatively, it has to be acknowledged that the low standardized mortality ratio for operatively treated elderly patients applies only to a selected population of patients who are fit for their age. Findings from previous literature support at least similar or even better survival in operatively treated patients.^10,14,22-25^ However, even if conservatively treated patients were included in the current study, in a register-based setting a comparison between operative and conservative treatment would not be justified, since the choice of treatment is made based on the type and severity of the injury as well as patient-specific factors. All in all, our results suggest that with proper patient selection surgery seems to be a valid and relatively safe treatment option for cervical fractures even in patients aged 85 years or older.

In the regression analysis we found that age and spinal cord injury were predictors of postoperative mortality. In a very recent study from the Swedish spine register a similar finding to that of ours was found.^20^ In the current study sex, the level of injury and surgery, opioid use and perioperative complications were not associated with mortality. In line with our findings, in literature age and spinal cord injury are consistently reported as the main predictors of mortality in larger population-based register studies.^10,19,21,26,27^

The observed complication rate of 3.8% was strikingly low in a cohort of trauma patients, which might suggest that register data may lack minor complications. However, it is noteworthy that mortality, being the main outcome of the study, was not included in this number. Furthermore, complications did not predict mortality.

We found that a considerable proportion of patients used opioids postoperatively after discharge. Opioid use did not predict mortality but, interestingly, opioid use was more common in patients who were alive at the end of the study period. We believe that this difference is explained by the setting in which the patients are treated after the initial hospital stay. Patients with a spinal cord injury, who have a higher risk of mortality, are likely to be transferred to a rehabilitation unit or to a nursing home. If the patient is treated at a healthcare facility, the medication is administered by the facility and not purchased by the patient, and therefore the medication is not recorded in the national prescription database. In brief, more severely injured patients, who are more likely to die, do not purchase their medication themselves, which explains the seemingly higher consumption of opioid in the patients who are alive.

In the current study, all types of operatively treated cervical spine fractures were included and analysed combined. This can be justified by several previous studies reporting comparable mortality rates for patients with upper as with subaxial cervical injuries.^20,21,28^ The inclusion of various types of injury in our study brings along variation in the applied surgical strategies. However, the surgical strategy does not seem to come up as a predictor of outcome, since it is selected based on the type of pathology.^21^ In the current study, the use of a posterior surgical approach was associated with an increased risk of mortality in the regression analysis. However, this finding needs to be interpreted with caution, since albeit being a less invasive approach, anterior surgery is usually applied for more benign injuries, whereas more severe injuries warrant a posterior or 360-degree approach.^29,30^ A 360-degree approach is likely to be preferred over a posterior approach in high-demand patients with better general health, which may explain that the 360-degree approach was not associated with increased mortality. All in all, the choice of the surgical approach is not only made based on the injury but also influenced by the general health of the patient. Therefore, mortality related to the surgical approach is likely to represent only a surrogate marker of injury severity and comorbidity, which does not imply causality.

The main drawback of the FinSpine register is that it includes only operatively treated patients and does not provide data on the outcome of conservative treatment. FinSpine was implemented during the study period with increasing coverage towards the end of it. Even if currently all centers, which operate spine are contributing to the register, there were some missing data on the level of surgery, which led to the exclusion of these patients (Figure 1). In emergency surgery, patient-reported data are also inherently incomplete, and therefore not applicable in the current study. Furthermore, while FinSpine records the level of injury, it does not include radiological images or provide details on the specific type of injury, eg, by the AOSpine classification.^31,32^ The definition of spinal cord injury is recorded as a binary variable in FinSpine according to the diagnosis made by the operating surgeon based on the presence of a clinically relevant paresis or paralysis. However, since the severity of the spinal cord injury is not recorded, both complete and incomplete injuries are analysed combined. Outcome data on mortality was available for all patients, but the low completion rate of patient-reported outcome measures and limitations in some other baseline and follow-up data prevents their use for more in-depth analyses and remains as a drawback of this study.

To conclude, nationwide population-based FinSpine register data shows that age and spinal cord injury are independent risk factors for mortality after surgery for cervical spine fractures. Patients had excess mortality as compared to the general population and the mortality increased with age. Even if mortality was high in elderly patients, the low SMR, representing excess mortality compared to the general population of the same age, supports the role of operative management with proper patient selection even in advanced age.

## Data Availability

National register data is curated by the Finnish Institute for Health and Welfare and cannot be publicly shared by the authors.*
